# Supplementary material for: Use of General Health Examination and Cancer Screening among People with Disability Who Need Support from Others: Analysis of the 2016 Comprehensive Survey of Living Conditions in Japan
Source: Int J Environ Res Public Health. 2024 Feb 13;21(2):219. doi: 10.3390/ijerph21020219 (PMC10888656; doi:10.3390/ijerph21020219)
Supplement: Supplementary file 1 [file ijerph-21-00219-s001.zip › 2.manuscript_revised_S1.pdf]

Table S1. The relevant original questionnaire items (in Japanese) and their English version (translated by the authors) in the Comprehensive Survey of Living Conditions of Japan in 2016.

Demographic variables

2016 年国民生活基礎調査		Comprehensive Survey of Living Conditions in 2016	
質問	選択肢	Questionnaire items	Answer option
性		Sex	
	男		Male
	女		Female
出生年月		Month of birth	
	年		Year
	月		Month
教育		Educational qualification	
	小学・中学		Primary and junior high school
	高校・旧性中		High school
	専門学校		Vocational school
	短大・高専		Community college or technical college
	大学		University
	大学院		Post graduate school
配偶者の有無		Marital status	
	配偶者あり		Married
	未婚		Single
	死別		Widowed
	離別		Divorced

7 *Physiological variables*

2016年国民生活基礎調査		Comprehensive Survey of Living Conditions in 2016	
質問	選択肢	Questionnaire items	Answer option
あなたは現在、傷病（病気やけが）で病院や診療所（医院、歯科医院）、 あんま・はり・きゅう・柔道整復師（施術所）に通っていますか?		Do you, currently, have a constant visit to hospital, clinic, acupuncture, moxibustion, Japanese massage, or Judo therapy for your health conditions (diseases or injuries)? †	
	通っている		Yes, I do
	通っていいない		No, I do not
あなたの現在の健康状態はいかがですか?		What do you think of your general health condition?	
	よい		Very good
	まあよい		Good
	ふつう		Not good not bad
	あまりよくない		Bad
	よくない		Very bad
a)あなたは週に何日くらいお酒(清酒、焼酎、ビール、洋酒など)を飲みますか?		a) How often do you drink alcohol? † †	
	毎日		Every day
	週 5-6 日		Five to six days per week
	週 3-4 日		Three to four days per week
	週 1-2 日		One to two days per week
	月 1-3 日		One to three days per a month
	ほとんど飲まない		I seldom drink alcohol
	やめた		I quit drinking alcohol

	飲まない		I do not drink alcohol
b)お酒を飲む日は１日あたり、どのくらいの量を飲みますか。		b）How much alcohol do you drink in a day? ††	
	１合（180ml）未満		Less than 180 ml
	１合以上２合（360ml）未満		180ml ≦ alcohol drink < 360ml
	２合以上３合（540ml）未満		360ml ≦ alcohol drink < 540ml
	３合以上４合（720ml）未満		540ml ≦ alcohol drink < 720ml
	４合以上５合（900ml）未満		720ml ≦ alcohol drink < 900ml
	５合（900ml）以上		900ml ≦ alcohol drink
あなたはたばこを吸いますか？		Do you smoke?	
	毎日吸っている		Every day
	時々吸う日がある		Sometimes
	以前は吸っていたが１か月以上吸っていない		I quit smoking for more than a month
	吸わない		I do not smoke

† No clear definition regarding the frequency of visit is provided in the questionnaire.

†† There are two kinds of questions regarding alcohol consumptions: a) frequency and b) amount of drinking. For calculating the amount of ethyl alcohol consumptions (g) per week, we equivalized 180ml alcohol drink with 20g ethyl alcohol. Furthermore, we interpreted each answer option as followed:

Question a)(frequency): every day; 7 days/week, five to six days per week; 6 days/week, three to four days per week; 4 days/week; one to two days per week; 2 days/week; one to three days per a month; 0.7 days/week and, I seldom drink alcohols, or I quit drinking alcohols or I do not drink alcohols; 0 day/week

Question b) (amount): less than 180 ml; 90 ml/day, 180ml ≦ alcohols < 360ml; 270ml/day, 360ml ≦ alcohols < 540ml; 450ml/day, 540ml ≦ alcohols < 720ml; 630ml/day, 720ml ≦ alcohols < 900ml; 810ml/day and, 900ml ≦ alcohols; 990ml/day

We calculated weekly ethyl alcohol consumption by multiplying the frequency and amount of alcohol consumption.

2016 年国民生活基礎調査		Comprehensive Survey of Living Conditions in 2016	
質問	選択肢	Questionnaire items	Answer option
現在の暮らしの状況を総合的にみて、どう感じていますか。		What do you think your current general economic situation?	
	大変苦しい		Very poor
	やや苦しい		Poor
	普通		Not poor not wealthy
	ややゆとりがある		Wealthy
	大変ゆとりがある		Very wealthy
次の(ｱ)から(ｶ)の質問について、過去 1 か月の間どうでしたか？<br><br>ア)神経過敏に感じましたか<br>イ）絶望的だと感じましたか<br>ウ）そわそわ、落ち着かなく感じましたか<br><br>エ）気分が沈み込んで、何が起こっても気が　晴れないように感じましたか<br>オ）何をするのも骨折りだと感じましたか<br><br>カ）自分は価値のない人間だと感じましたか		The following questions are about how you have been feeling during the past 30 days.<br>1) How often did you feel nervous?<br>2) How often did you feel hopeless?<br>3) How often did you feel restless or fidgety?<br>4) How often did you feel so depressed that nothing could cheer you up?<br>5) How often did you feel everything was an effort?<br>6) How often did you feel worthless?	
	いつも		All

	たいてい		Most
	ときどき		Some
	少しだけ		A little
	まったくない		None
医療保険の加入状況		Health insurance	
	国民健康保険（市町村か組合）		National Health Insurance
	被用者保険（加入者本人か家族（被扶養者））		Employee insurance
	後期高齢者医療制度		Medical care system for older adults in the latter stage of life (over 75 years)
	その他		Other
a)仕事の状況††		Working situation	
	仕事あり（主に仕事をしている、主に家事で仕事あり、主に通学で仕事あり、その他）		Working (mainly working, housework, schooling or others)
	仕事なし（通学、家事、その他）		Not-working (housework, schooling or others)
b)勤めか自営かの別††		Types of work	
	一般常勤者（契約期間の定めのない雇用者）		1)Employee (contract period: permanent)
	一般常勤者（契約期間が 1 か月以上の雇用者）		2) Employee (contract period: more than a month)
	1 月以上 1 年未満の契約の雇用者		3) Contract employee(1 month ≦ contract period ≦ 1 year)
	日々又は 1 月未満の契約の雇用者		4) Contract employee (contract period < 1 month)
	会社・団体等の役員		5) Executive of company or corporation

自営業者（雇人あり）	6) Self-employed (with employee)
自営業者（雇人なし）	7) Self-employed (without employee)
家族従業者（自家営業の手伝い）	8) Family worker
内職	9) Doing piecework at home
その他	10) Other

† † There are two questions regarding employment status: a) working situation and b) types of work. We considered employment status as:

Unemployed : a) Answer; Not-working

Employed : a) Answer; Working AND b) Answer; 1–4)

Self- employed : a) Answer; Working AND b) Answer; 6 or 7)

Employed(others): a) Answer; Working AND b) Answer; 5 or 8–10)
