# Supplementary material for: Use of General Health Examination and Cancer Screening among People with Disability Who Need Support from Others: Analysis of the 2016 Comprehensive Survey of Living Conditions in Japan
Source: Int J Environ Res Public Health. 2024 Feb 13;21(2):219. doi: 10.3390/ijerph21020219 (PMC10888656; doi:10.3390/ijerph21020219)
Supplement: Supplementary file 1 [file ijerph-21-00219-s001.zip › 3.manuscript_revised_S2.pdf]

**Table S2.** The number and percentage of data with missing value among data which met the inclusion criteria of age (20 ≦ years old ≦ 75) and living place (house). (N=10283; men=4891, women=5302). These data were removed from analysis.

| | Number of data with missing value | ( | % | ) |
|---|---|---|---|---|
| Outcome variables | | | | |
| General health examination | 90 | ( | 0.88 | ) |
| Lung cancer screening | 303 | ( | 2.95 | ) |
| Colorectal cancer screening | 327 | ( | 3.18 | ) |
| Gastric cancer screening | 280 | ( | 2.72 | ) |
| Cervical cancer screening † | 151 | ( | 2.85 | ) |
| Breast cancer screening † | 137 | ( | 2.58 | ) |
| Explanatory variables | | | | |
| Disability | 0 | ( | 0 | ) |
| Cofounding variables | | | | |
| Sex | 0 | ( | 0 | ) |
| Age | 0 | ( | 0 | ) |
| Marital status | 0 | ( | 0 | ) |
| Educational qualification | 1401 | ( | 13.62 | ) |
| Constant visit to hospitals | 69 | ( | 0.67 | ) |
| Subjective health status | 98 | ( | 0.95 | ) |
| Alcohol consumption | 152 | ( | 1.48 | ) |
| Smoking habit | 119 | ( | 1.16 | ) |
| Subjective financial state | 0 | ( | 0 | ) |
| Kessler Psychological Distress Scale | 325 | ( | 3.16 | ) |
| Health insurance | 124 | ( | 1.21 | ) |
| Employment status | 127 | ( | 1.24 | ) |

† Percentages were calculated for only women (n=5302)
