# Supplementary material for: Use of General Health Examination and Cancer Screening among People with Disability Who Need Support from Others: Analysis of the 2016 Comprehensive Survey of Living Conditions in Japan
Source: Int J Environ Res Public Health. 2024 Feb 13;21(2):219. doi: 10.3390/ijerph21020219 (PMC10888656; doi:10.3390/ijerph21020219)
Supplement: Supplementary file 1 [file ijerph-21-00219-s001.zip › 4.manuscript_revised_S3.pdf]

Table S3-A and S3-B

The results of the binomial logistic regression analysis that used all variables in forced entry method.

Table S3-A. Unadjusted and adjusted odds ratio for non-participation in the general health examination and cancer screening.

	General health examination (*n*=8,438)		Lung cancer screening (*n*=6,042)		Colorectal cancer screening (*n*=6,030)	
	Unadjusted OR (95%CI)	Adjusted OR (95%CI)	Unadjusted OR (95%CI)	Adjusted OR (95%CI)	Unadjusted OR (95%CI)	Adjusted OR (95%CI)
Disability						
No-need any support or supervision	1 (Ref.)	1 (Ref.)	1 (Ref.)	1 (Ref.)	1 (Ref.)	1 (Ref.)
Need any support or supervision	2.81 (1.92,4.11)	1.73 (1.14,2.62)	2.30 (1.46,3.62)	1.56 (0.96,2.51)	2.26 (1.40,3.64)	1.78 (1.08,2.94)
Sex						
Male	1 (Ref.)	1 (Ref.)	1 (Ref.)	1 (Ref.)	1 (Ref.)	1 (Ref.)
Female	1.68 (1.53,1.86)	1.71 (1.51,1.93)	1.60 (1.44,1.77)	1.55 (1.37,1.76)	1.44 (1.30,1.60)	1.53 (1.36,1.74)
Age (years)						
65–74	1 (Ref.)	1 (Ref.)	1 (Ref.)	1 (Ref.)	1 (Ref.)	1 (Ref.)
40–64	0.65 (0.57,0.73)	1.38 (1.18,1.61)	0.89 (0.79,0.99)	1.40 (1.2,1.62)	0.95 (0.85,1.06)	1.22 (1.05,1.41)
20–39	0.93 (0.82,1.07)	2.13 (1.75,2.6)	− (-)	− (-)	− (-)	− (−)
Marital status						
Married	1 (Ref.)	1 (Ref.)	1 (Ref.)	1 (Ref.)	1 (Ref.)	1 (Ref.)
Single	1.37 (1.22,1.53)	1.37 (1.18,1.58)	1.50 (1.27,1.78)	1.47 (1.22,1.76)	1.72 (1.44,2.05)	1.64 (1.36,1.98)
Divorced/widowed	1.35 (1.14,1.59)	1.04 (0.86,1.25)	1.38 (1.17,1.63)	1.10 (0.92,1.31)	1.52 (1.28,1.80)	1.20 (1,1.43)
Educational qualification						
Vocational school/junior college/community(technical) college/university/post graduate school	1 (Ref.)	1 (Ref.)	1 (Ref.)	1 (Ref.)	1 (Ref.)	1 (Ref.)
High school	1.47 (1.32,1.63)	1.33 (1.19,1.49)	1.43 (1.28,1.59)	1.22 (1.09,1.37)	1.52 (1.37,1.70)	1.33 (1.19,1.5)
Primary/junior high school	2.71 (2.30,3.20)	2.02 (1.67,2.45)	1.90 (1.59,2.27)	1.41 (1.16,1.72)	2.27 (1.89,2.73)	1.82 (1.48,2.23)
Subjective financial state						
Wealthy	1 (Ref.)	1 (Ref.)	1 (Ref.)	1 (Ref.)	1 (Ref.)	1 (Ref.)
Not poor not wealthy	1.34 (1.04,1.71)	1.17 (0.9,1.52)	1.59 (1.25,2.01)	1.44 (1.12,1.84)	1.63 (1.29,2.05)	1.43 (1.13,1.82)

Poor	1.99 (1.56,2.54)	1.62 (1.25,2.1)	2.25 (1.78,2.83)	1.91 (1.49,2.44)	2.39 (1.90,2.99)	1.92 (1.52,2.44)
Health insurance						
Employee insurance	1 (Ref.)	1 (Ref.)	1 (Ref.)	1 (Ref.)	1 (Ref.)	1 (Ref.)
National Health Insurance	2.46 (2.22,2.72)	1.66 (1.45,1.91)	1.78 (1.60,1.98)	1.38 (1.19,1.59)	1.50 (1.35,1.67)	1.25 (1.08,1.45)
Other	4.33 (3.22,5.83)	2.94 (2.11,4.09)	3.45 (2.44,4.90)	2.53 (1.74,3.68)	2.87 (2.00,4.11)	2.06 (1.4,3.03)
Employment status						
Employed	1 (Ref.)	1 (Ref.)	1 (Ref.)	1 (Ref.)	1 (Ref.)	1 (Ref.)
Self-employed	3.14 (2.61,3.79)	3.54 (2.83,4.41)	1.86 (1.54,2.26)	1.98 (1.58,2.48)	1.58 (1.30,1.92)	1.69 (1.34,2.12)
Employed(other)	2.32 (1.92,2.79)	2.54 (2.07,3.13)	1.40 (1.16,1.69)	1.44 (1.17,1.76)	1.17 (0.97,1.41)	1.21 (0.99,1.48)
Unemployed	3.37 (3.02,3.76)	3.15 (2.75,3.62)	2.13 (1.90,2.40)	2.00 (1.72,2.32)	1.50 (1.33,1.68)	1.37 (1.18,1.6)
Kessler Psychological Distress Scale						
Normal (total score =< 4)	1 (Ref.)	1 (Ref.)	1 (Ref.)	1 (Ref.)	1 (Ref.)	1 (Ref.)
Mild illness (5 ≦ total score ≦ 12)	1.17 (1.05,1.31)	1.11 (0.98,1.26)	1.16 (1.03,1.31)	1.08 (0.95,1.23)	1.22 (1.08,1.37)	1.15 (1.01,1.31)
Severe illness (13 ≦ total score)	1.36 (1.07,1.74)	1.08 (0.82,1.42)	1.40 (1.04,1.89)	1.09 (0.79,1.51)	1.41 (1.04,1.91)	1.17 (0.84,1.62)
Constant visit to hospitals†						
Yes (constant visit)	1 (Ref.)	1 (Ref.)	1 (Ref.)	1 (Ref.)	1 (Ref.)	1 (Ref.)
No (no-constant visit)	1.22 (1.11,1.35)	1.57 (1.39,1.76)	1.26 (1.14,1.39)	1.49 (1.33,1.67)	1.39 (1.26,1.54)	1.57 (1.4,1.76)
Subjective health status						
Good	1 (Ref.)	1 (Ref.)	1 (Ref.)	1 (Ref.)	1 (Ref.)	1 (Ref.)
Normal	1.04 (0.94,1.16)	1.00 (0.89,1.12)	1.05 (0.94,1.17)	1.01 (0.9,1.14)	1.05 (0.94,1.17)	1.01 (0.9,1.14)
Bad	1.37 (1.17,1.61)	1.17 (0.97,1.42)	1.35 (1.14,1.60)	1.17 (0.96,1.42)	1.27 (1.07,1.50)	1.12 (0.92,1.36)
Alcohol consumption						
Never or quit drinking	1 (Ref.)	1 (Ref.)	1 (Ref.)	1 (Ref.)	1 (Ref.)	1 (Ref.)
Social drinker/low-risk group (> 0 to ≤ 100 g/week)	0.66 (0.58,0.74)	0.80 (0.7,0.91)	0.65 (0.57,0.74)	0.78 (0.68,0.89)	0.66 (0.58,0.75)	0.76 (0.66,0.87)
Middle- risk drinking (> 100 to ≤ 350 g/week)	0.57 (0.50,0.66)	0.80 (0.68,0.94)	0.61 (0.54,0.70)	0.81 (0.7,0.94)	0.71 (0.62,0.81)	0.88 (0.76,1.02)
High-risk drinking (> 350 g/week)	0.80 (0.60,1.07)	1.06 (0.77,1.45)	0.88 (0.65,1.18)	1.05 (0.77,1.45)	0.77 (0.57,1.04)	0.81 (0.59,1.12)

Smoking habit						
Never/ex-smoker	1 (Ref.)	1 (Ref.)	1 (Ref.)	1 (Ref.)	1 (Ref.)	1 (Ref.)
Current smoker	1.09 (0.97,1.22)	1.32 1.15,1.51)	1.13 1.00,1.27)	1.28 1.12,1.47)	1.40 1.24,1.59)	1.51 1.32,1.74)
Model chi square test	P<0.001		P<0.001		P<0.001	
Hosmer-Lemeshow test	P=0.384		P=0.064		P=0.911	

Note: OR, odds ratio; CI, confidence interval; Ref., reference

† No clear definition regarding the frequency of visit is provided in the questionnaire.

Table S3-B Unadjusted and adjusted odds ratio for non-participation in the cancer screening.

	Gastric cancer screening (*n*=4,318)		Cervical cancer screening (*n*=4,261)		Breast cancer screening (*n*=3,098)	
	Unadjusted OR (95%CI)	Adjusted OR (95%CI)	Unadjusted OR (95%CI)	Adjusted OR (95%CI)	Unadjusted OR (95%CI)	Adjusted OR (95%CI)
Disability						
No-need any support or supervision	1 (Ref.)	1 (Ref.)	1 (Ref.)	1 (Ref.)	1 (Ref.)	1 (Ref.)
Need any support or supervision	2.71 (1.56,4.70)	2.27 (1.27,4.05)	3.00 (1.54,5.85)	2.12 (1.04,4.32)	2.74 (1.33,5.62)	2.22 (1.04,4.72)
Sex						
Male	1 (Ref.)	1 (Ref.)	- (-)	− (-)	- (-)	− (-)
Female	1.53 (1.35,1.72)	1.59 (1.37,1.85)	- (-)	− (-)	- (-)	− (-)
Age (years)						
65–74	- (-)	− (-)	1 (Ref.)	1 (Ref.)	1 (Ref.)	1 (Ref.)
40–64	- (-)	− (-)	0.44 (0.38,0.52)	0.57 (0.46,0.69)	0.50 (0.43,0.59)	0.66 (0.53,0.81)
20–39	- (-)	− (-)	0.65 (0.54,078)	0.53 (0.41,0.68)	- (-)	− (-)
65–74	1 (Ref.)	1 (Ref.)	- (-)	− (-	- (-)	− (-
50–64	0.88 (0.78,0.99)	1.22 (1.04,1.43)	- (-)	− (-	- (-)	− (-
Marital status						
Married	1 (Ref.)	1 (Ref.)	1 (Ref.)	1 (Ref.)	1 (Ref.)	1 (Ref.)
Single	1.62 (1.28,2.04)	1.58 (1.23,2.02)	2.59 (2.18,3.07)	3.37 (2.76,4.13)	1.30 (0.98,1.72)	1.55 (1.15,2.08)
Divorced/widowed	1.53 (1.27,1.84)	1.22 (1,1.49)	1.61 (1.33,1.96)	1.27 (1.03,1.56)	1.53 (1.25,1.88)	1.25 (1,1.55)
Educational qualification						
Vocational school/junior col-	1 (Ref.)	1 (Ref.)	1 (Ref.)	1 (Ref.)	1 (Ref.)	1 (Ref.)

lege/community(technical) college/university/post graduate school						
High school	1.51 (1.33,1.72)	1.27 (1.11,1.46)	1.30 (1.15,1.48)	1.20 (1.04,1.38)	1.61 (1.38,1.87)	1.32 (1.12,1.55)
Primary/junior high school	2.05 (1.68,2.50)	1.50 (1.2,1.87)	2.86 (2.21,3.72)	1.87 (1.4,2.5)	3.10 (2.36,4.07)	1.80 (1.33,2.44)
Subjective financial state						
Wealthy	1 (Ref.)	1 (Ref.)	1 (Ref.)	1 (Ref.)	1 (Ref.)	1 (Ref.)
Not poor not wealthy	2.05 (1.55,2.70)	1.76 (1.32,2.35)	1.60 (1.21,2.12)	1.43 (1.07,1.92)	1.97 (1.41,2.75)	1.82 (1.28,2.57)
Poor	2.84 (2.16,3.73)	2.29 (1.72,3.06)	2.26 (1.72,2.98)	2.00 (1.5,2.68)	2.69 (1.94,3.74)	2.41 (1.7,3.4)
Health insurance						
Employee insurance	1 (Ref.)	1 (Ref.)	1 (Ref.)	1 (Ref.)	1 (Ref.)	1 (Ref.)
National Health Insurance	1.74 (1.54,1.96)	1.40 (1.19,1.64)	1.82 (1.59,2.09)	1.36 (1.15,1.61)	1.85 (1.59,2.15)	1.27 (1.05,1.53)
Other	3.36 (2.26,4.98)	2.24 (1.47,3.42)	5.14 (2.83,9.34)	3.02 (1.62,5.63)	6.08 (3.17,11.7)	3.33 (1.68,6.59)
Employment status						
Employed	1 (Ref.)	1 (Ref.)	1 (Ref.)	1 (Ref.)	1 (Ref.)	1 (Ref.)
Self-employed	1.66 (1.33,2.07)	1.67 (1.3,2.16)	1.30 (0.88,1.93)	1.11 (0.73,1.69)	1.61 (1.06,2.45)	1.36 (0.86,2.13)
Employed(other)	1.43 (1.15,1.78)	1.47 (1.16,1.86)	1.23 (0.97,1.57)	1.23 (0.94,1.61)	1.55 (1.20,2.01)	1.40 (1.05,1.86)
Unemployed	1.78 (1.55,2.03)	1.57 (1.31,1.86)	1.39 (1.22,1.58)	1.23 (1.05,1.44)	1.72 (1.48,2.00)	1.39 (1.16,1.68)
Kessler Psychological Distress Scale						
Normal (total score =< 4)	1 (Ref.)	1 (Ref.)	1 (Ref.)	1 (Ref.)	1 (Ref.)	1 (Ref.)
Mild illness (5 ≦ total score ≦ 12)	1.19 (1.03,1.37)	1.13 (0.97,1.32)	0.97 (0.85,1.12)	0.94 (0.81,1.1)	0.92 (0.78,1.09)	0.90 (0.75,1.08)
Severe illness (13 ≦ total score)	1.11 (0.76,1.61)	0.83 (0.56,1.25)	1.01 (0.75,1.36)	0.86 (0.61,1.2)	0.97 (0.66,1.43)	0.87 (0.57,1.33)
Constant visit to hospitals†						
Yes (constant visit)	1 (Ref.)	1 (Ref.)	1 (Ref.)	1 (Ref.)	1 (Ref.)	1 (Ref.)
No (no-constant visit)	1.38 (1.22,1.56)	1.56 (1.36,1.78)	1.20 (1.06,1.35)	1.53 (1.33,1.77)	1.27 (1.10,1.46)	1.68 (1.44,1.98)
Subjective health status						
Good	1 (Ref.)	1 (Ref.)	1 (Ref.)	1 (Ref.)	1 (Ref.)	1 (Ref.)
Normal	1.09 (0.96,1.25)	1.06 (0.92,1.23)	1.20 (1.05,1.37)	1.16 (1.01,1.34)	1.09 (0.93,1.27)	1.04 (0.88,1.23)
Bad	1.24 (1.02,1.51)	1.11 (0.88,1.39)	1.16 (0.94,1.42)	1.13 (0.89,1.44)	1.11 (0.88,1.41)	1.09 (0.83,1.42)

Alcohol consumption												
Never or quit drinking	1	(Ref.)	1	(Ref.)	1	(Ref.)	1	(Ref.)	1	(Ref.)	1	(Ref.)
Social drinker/low-risk group (> 0 to ≤ 100 g/week)	0.65	(0.56,0.75)	0.77	(0.65,0.9)	0.85	(0.74,0.99)	0.92	(0.79,1.08)	0.72	(0.61,0.86)	0.81	(0.68,0.98)
Middle- risk drinking (> 100 to ≤ 350 g/week)	0.70	(0.60,0.82)	0.92	(0.77,1.09)	0.72	(0.58,0.90)	0.82	(0.65,1.04)	0.92	(0.71,1.18)	1.05	(0.8,1.38)
High-risk drinking (> 350 g/week)	0.90	(0.61,1.32)	1.10	(0.73,1.65)	0.76	(0.43,1.35)	0.81	(0.44,1.5)	0.89	(0.44,1.81)	1.12	(0.53,2.34)
Smoking habit												
Never/ex-smoker	1	(Ref.)	1	(Ref.)	1	(Ref.)	1	(Ref.)	1	(Ref.)	1	(Ref.)
Current smoker	1.37	(1.18,1.60)	1.55	1.31,1.84)	1.37	1.12,1.69)	1.37	1.1,1.71)	1.83	1.42,2.36)	1.81	1.39,2.36)
Model chi square test	P<0.001				P<0.001				P<0.001			
Hosmer-Lemeshow test	P=0.575				P=0.919				P=0.853			

Note: OR, odds ratio; CI, confidence interval; Ref., reference

† No clear definition regarding the frequency of visit is provided in the questionnaire.
